# Multi-level toxicity assessment of the antidepressant venlafaxine in embryos/larvae and adults of zebrafish (*Danio rerio*)

**DOI:** 10.1590/1678-4685-GMB-2022-0377

**Published:** 2023-09-08

**Authors:** Ana Clara Oliveira, Maria Luiza Fascineli, Paula Martins de Oliveira, Guilherme Martins Gelfuso, Rolando André Rios Villacis, Cesar Koppe Grisolia

**Affiliations:** 1 Universidade de Brasília (UnB), Instituto de Ciências Biológicas, Departamento de Genética e Morfologia, Laboratório de Genética Toxicológica (GTOX), Brasília, DF, Brazil.; 2 Universidade Federal da Paraíba (UFPB), Centro de Ciências da Saúde, Departamento de Morfologia (DMORF), João Pessoa, PB, Brazil.; 3 Universidade de Brasília (UnB), Faculdade de Ciências da Saúde, Laboratório de Tecnologia de Medicamentos, Alimentos e Cosméticos (LTMAC), Brasília, DF, Brazil.

**Keywords:** Danio rerio, venlafaxine, acute exposure, biomarkers, genotoxicity

## Abstract

The toxic effects of venlafaxine (VLX) on aquatic organisms have already been verified and therefore are a proven matter of concern. Herein, we evaluated zebrafish embryos/adults after acute exposure to VLX. Embryos/larvae were exposed to different concentrations of VLX (100-1000 mg/L; 1.33 as a dilution factor), to evaluate mortality/developmental changos and to analyze biomarkers (0.002-100 mg/L). For adults, mortality, genotoxicity, and biomarkers were assessed in five different concentrations of VLX (1-100 mg/L). The median lethal concentration (LC_50_-168h) was 274.1 mg/L for embryos/larvae, and >100 mg/L for adults (LC_50_-96h). VLX decreased the heart rate frequency and caused premature hatching and lack of equilibrium in embryos/larvae exposed to different concentrations ranging from 100 to 562.5 mg/L. The activity of acetylcholinesterase (AChE) was inhibited in larvae exposed to 1, 25 and 100 mg/L. Glutathione-S-transferase (GST) activity was reduced in both larvae and adults after exposure to different concentrations, mainly at 25 mg/L. For both larvae and adults, lactate dehydrogenase (LDH) activity increased after 100 mg/L of VLX exposure. No DNA damage was observed in peripheral erythrocytes. Exposure to VLX may cause adverse effects on zebrafish in their early and adult life stages, interfering with embryo-larval development, and can induce physiological disturbances in adults.

## Introduction

A wide range of organic and synthetic compounds, used in large quantities by modern society, reach aquatic ecosystems and negatively affect not only water quality for human consumption, but also the survival of aquatic life (Lapworth et al., 2012; Bundschuh et al., 2017). Based on sustainable, social and economic development, the potential harmful effects of these aquatic pollutants should be monitored. In fact, residues of psychiatric drugs have been consistently detected in aquatic ecosystems (Choi et al., 2018). Diverse toxic effects on aquatic biota, mainly detected in fish, have been associated with exposure to psychiatric drugs, including changes in survival rates, morphology, behaviour and gene expression (Valenti Jr et al., 2009; Marcon et al., 2016; de Farias et al., 2020; Oliveira et al., 2021).

Venlafaxine (VLX) is a selective serotonin noradrenaline reuptake inhibitor (SNRI) that acts by inhibiting neuronal serotonin/noradrenaline reuptake, increasing the amount of these neurotransmitters in the synaptic cleft (Silva and Fernández-Guasti, 2019). VLX is one of the main psychiatric drugs used in the treatment of depression, including depression with associated anxiety (Fong et al., 2015). In Brazil, 24,000 kg of VLX were sold in 2018 (Pivetta et al., 2020). The biological effects of VLX are related to its mechanism of action, increasing or blocking the reuptake of neurotransmitters, which in turn interferes with nervous transmission in the exposed organisms (Kar and Roy, 2012).

Due to its wide use and low breakdown in sewage treatment plants, VLX is considered a surface water contaminant, and its residues and metabolites are found in the aquatic environment of a number of countries. Schlüsener et al. (2015) detected up to 180 ng/L of VLX in Rhine River surface waters; while in the Douro River and Leça (Portugal), Fernandes et al. (2020) identified VLX dissolved in water and accumulated in sediments at concentrations of 641 ng/L and 0.251 ng/g, respectively, signaling this drug as a pollutant of high risk to the biodiversity of these ecosystems.

VLX bioaccumulates in the tissues of non-target organisms (Martínez-Morcillo et al., 2020). The authors found up to 6 ng/g of VLX in tissues from a bivalve mollusc (*Cerastoderma edule*), collected in the Atlantic Ocean (Martínez-Morcillo *et al*., 2020). Acute exposure to 20 µg/L of VLX decreased social activity and shoal cohesion in *Argyrosomus regius* fish (Maulvault et al., 2018). In zebrafish (*Danio rerio*), larva swimming activity was reduced by 40% after exposure to 100 µg/L of VLX (Huang et al., 2019). Additionally, zebrafish spawning decreases significantly after chronic exposure (6-week period) to a pharmaceutical mixture of acetaminophen, carbamazepine, gemfibrozil and VLX (Galus et al., 2013). This chemical mixture also caused alterations in the ovarian and kidney proximal tubule morphology of the exposed animals (Galus *et al*., 2013). Recently, changes in the expression of genes crucial to development (e.g., *pax6*-eyes and *bmp4*-bones) have been identified in *Danio rerio*/*Xenopus tropicalis* embryos exposed to 0.3 µg/L of VLX (Sehonova et al., 2019). 

Zebrafish present serotonergic and cholinergic systems, making them an excellent model organism for the study of substances that act on those systems, such as antidepressants (Panula et al., 2006; Yamamoto et al., 2011; Bambino and Chu, 2017). Currently, zebrafish are a widely accepted model for ecotoxicological studies. Despite the increasing number of studies showing the presence of psychiatric drugs in aquatic environments, their potential risks are not yet fully understood (Choi et al., 2018). An ecotoxicological approach based on different endpoints is needed to understand how such chemicals interact with aquatic organisms. These pharmaceuticals are stable in water and flow continuously into water bodies due to their wide usage, thus becoming persistent contaminants (Zuccato et al., 2005). 

In this context, the present study aimed to investigate VLX acute toxicity in zebrafish, searching for adverse effects through different endpoints in embryos/larvae and adults, including mortality, morphological changes, biomarkers, and genotoxicity.

## Material and Methods

### Chemical and HPLC analysis

Venlafaxine hydrochloride powdered reagent (≥ 98%) from Sigma-Aldrich was used in this study. The empirical formula is C_17_H_27_NO_2_.HCl. Previously, solutions of VLX in concentrations between 1 and 25 mg/L had been maintained in the water under the same experimental conditions as the toxicity tests, to evaluate its breakdown in the water. To evaluate VLX stability in water, sample solutions were analysed daily for a period of seven days using High Performance Chromatography (HPLC Prominence, Shimadzu, Kyoto, Japan), following the methods reported by Shen et al. (2018). The standard curve was determined by High Performance Chromatography (HPLC, Shimadzu-Prominence, Kyoto, Japan) coupled to degasser (model DGU 20A_5_), solvent distribution module (model LC - 20AT), automatic sampler (model SIL - 20 AHT), column heater (model CTO - 20A), UV-VIS detector (model SPD-20A) and controller CBM-20A. The column used was C-18 reverse phase CLC - ODS (M) (4,6 mm i.d X 150 mm, 5μm). VLX solutions in concentrations between 1 and 25 mg/L were prepared in ultrapure water for standard curve determination (Figure S1). The gradient method was used with mobile phase composed of water containing sodium dihydrogen phosphate (0.05 mol/L) (A) and acetonitrile (72:28) (B). The following parameters were employed: flow rate of 0.5 mL/min, column temperature of 30 °C, fluorescence excitation wavelength of 276 nm and an emission wavelength of 598 nm. We used the software LC solution (Shimadzu, Tokyo, Japan) for data analysis and parameter determination. The percentage recovery analysis was performed using a stock solution of 16 mg/L of VLX, an intermediate concentration between those used to construct the standard curve (Table S1).

### Test organisms

Adult zebrafish were cultivated in a recirculating system (ZebTec housing system, Tecniplast, Buguggiate, Varese, Italy) at the Department of Genetics and Morphology, University of Brasília (UnB), Brazil. The procedures adopted for fish care and maintenance have been previously described (Oliveira et al., 2021). All tests were carried out following the International Guidelines for Biomedical Research Involving Animals, and our study was approved by the UnB Ethics Committee under Protocol n. 100226/2014.

### Embryo-larval exposure

*Fish Embryo Toxicity test*

Zebrafish eggs were obtained by breeding of fish in the Ispawn breeding system (Tecniplast). The day prior to breeding, males and females were sequentially added to the system and kept separated by a divider, in a proportion of two males for one female. Early in the morning, the divider was removed, and the spawning platform was lifted to initiate the spawning. The eggs were collected immediately after natural mating, rinsed in water, and checked using a stereomicroscope (Stemi, Carl Zeiss, Jena, Germany). The unfertilized eggs (<20%) and those with cleavage irregularities or injuries were discarded.

The fish embryo toxicity (FET) test was conducted according to the OECD (Organization for Economic Co-operation and Development) guideline Protocol 236 (OECD, 2013), with adaptations described by Oliveira et al. (2021). Based on the results of pre-tests, zebrafish embryos were exposed to eight different VLX concentrations (100, 177.9, 237.7, 316.4, 421.8, 562.5, 750 and 1000 mg/L). The test started up to 90 minutes after fertilization and continued for 168 h in a climate chamber (SL-24, Solab, Piracicaba, SP, Brazil) with controlled abiotic conditions (12:12 photoperiod - light and dark; 27 ±1 °C; pH of 7.5 ± 0.5). To ensure water quality and the presence of the VLX molecule during the entire exposure period test, test-solutions were renewed once after 96 h. Embryos and larvae were observed daily under a stereomicroscope (manual evaluation) and, before hatching, the following parameters were observed: egg coagulation, lack of otolith formation, general delay in development, lack of eye and/or body pigmentation, lack of somite formation, lack of heartbeat, oedemas and lack of hatching. After hatching, we evaluated spine malformation, oedema, swim bladder inflation and lack of equilibrium (embryos side-lying on the bottom of the microplate well after mechanical stimulus). The parameters were quantified as observed or not observed. Specifically, we also evaluated heart rate by manual counting of the number of beats/15s. FET test was performed in independent triplicates. For each concentration/external control, three different plates were used (experimental triplicate) with 24 individuals in each (one individual per well). Four wells for internal plate control and the others for exposure. Therefore, for each replicate, 20 individuals were analysed.

*Analysis of biomarkers in larvae*

We evaluated enzymes that act as biomarkers of neurotoxicity (acetylcholinesterase, AChE), cell detoxification (glutathione S-transferase, GST) and energy metabolism (lactate dehydrogenase, LDH). The activity of these enzymes was quantified after VLX acute exposure (168 h) to four sub-lethal concentrations (0.002, 1, 25, 100 mg/L), which were selected according to the FET test result. The experiments were performed with 250 eggs and 500 mL of test solution. We considered independent triplicates and, for each replicate, 10 pools of 15 viable larvae per concentration were collected. Protein extraction/quantification was performed as previously described (Oliveira et al., 2021). The activity of proteins was measured in quadruplicates (technical replicates) using a spectrophotometer (SpectraMax M2 microplate reader, Molecular Devices, Sunnyvale, CA, USA).

AChE activity was determined using acetylthiocholine (ACh) as the substrate, measuring the conjugation product between thiocholine (result of the degradation of ACh) and 5,5-dithiobis-2-nitrobenzoic acid (DTNB) (absorbance increase) at 414 nm, every 20 s, for 5 min, according to the method previously described by Ellman et al. (1961). GST activity was determined by measuring the conjugation product between glutathione and 1-Chloro-2,4-dinitrobenzene (CDNB) (absorbance increase) at 340 nm, every 40 s, for 5 min, according to the method of Habig and Jakoby (1981). Lastly, LDH activity was determined by measuring the reduction of pyruvate (substrate) and the oxidation of NADH at 340 nm, every 40 s, for 5 min, according to the method of Vassault (1983).

### Adult exposure

*Acute test*

The experiment was performed following the standard protocol of OECD 203 (OECD, 2019). Zebrafish adults of similar ages (~6 months old) were weighed and randomly distributed (in ratio: 1 male/1 female) in tanks (3 tanks per treatment, 7 animals per tank) containing 4 L of test solutions, thus representing independent triplicates. Based on previous pre-tests and FET assay concentrations, adults were exposed for 96 hours to five VLX concentrations (1, 25, 50, 75 and 100 mg/L) in a static condition. Fish mortality was monitored daily (every 24 h) for the 96 hours of the experiment. At the end of each test, the surviving animals were used to perform the enzyme activity assays and genotoxic analyses.

*Analysis of biomarkers in adults*

The same biomarkers tested in larvae (AChE, GST and LDH) were also evaluated in adults. After the acute exposure (96 h), 10 head and muscle samples per concentration were dissected and collected in microtubes with 0.5 ml of K-phosphate buffer (0.1 M, pH 7.4), frozen in liquid nitrogen and immediately stored at -80 ºC until the day of analysis. The following steps for quantification of enzymatic activity were identical to those described for larvae.

*Genotoxicity evaluation*

This analysis was performed at the same concentrations of VLX (1, 25, 50, 75 and 100 mg/L) and with the same animals used in the biochemical tests. The blood of animals was collected (1 µL) with a heparinized syringe and stored in microtubes containing 500 μL of fetal bovine serum (FBS). Of this mixture (FBS-blood), 40 and 50 μL were used to conduct the comet assay and the micronucleus/nuclear abnormalities test, respectively.

*Comet assay*

The alkaline comet assay was performed according to the procedure described by Kosmehl et al. (2008), with minor modifications. Forty µL of blood were mixed with 120 µL of 15% low melting point agarose prepared in phosphate buffered saline at 37**º**C in a water bath. This mixture was spread on a microscope slide precoated with normal agarose (5%). Slides were incubated for 120 min in a lysing solution (pH 10) at 4**º**C in the dark. The slides were placed in an electrophoresis buffer (pH > 13), and electrophoresis proceeded at 20 V and 300 mA for 15 min. The slides were washed three times with a neutralization buffer (0.4 M Tris-HCl, pH 7.5) for 5 min and dried in absolute ethanol. The slides were stained with ethidium bromide (20 µg/mL) and analysed using a fluorescence microscope (ZEISS Axioskop 2-HAL100, at 400x magnification) and the Comet IV Lite v 4.3 software (Perceptive Instruments, Suffolk, UK). The percentage of DNA in the tail (% DNA in tail) was measured in 200 nucleoids of each fish (100 per slide, 2 slides per animal). The values of % DNA in the tail were then classified in five categories of DNA damage: category 0 (no damage, 0-1% DNA in tail), category 1 (low damage, >1-25% DNA in tail), category 2 (medium damage, >25-45% DNA in tail), category 3 (high damage, >45-70% DNA in tail) and category 4 (very high damage, >70% DNA in tail) (García et al., 2011).

*Micronucleus and nuclear abnormalities test*

Fifty microliters of blood was smeared on clean glass slides, dried at room temperature, fixed in methanol 100% for 10 min and stained by Giemsa (5%). The slides were evaluated under a blind code; 3000 erythrocytes were microscopically scored for each sample at 1000X of magnification (1500 erythrocytes per slide, 2 slides per animal). The criteria for the identification of micronucleated erythrocytes (MN) of fish were: area smaller than one-third of the main nucleus; no connection with the main nucleus; no refraction and same colour and intensity as in the main nucleus (Fenech et al., 2003; Barsiene et al., 2006). Erythrocytes were also scored to classify nuclear abnormalities (NAs), of which the most common were binucleated cell, nuclear bud, blebbed nucleus, lobed nucleus and notched nucleus (Carrasco et al., 1990; Palhares and Grisolia, 2002).

### Statistical analyses

Statistical analyses were performed using the software Sigma Plot 12.5 (Systat Software, San Jose, CA, USA). Lethal and effective concentrations (LC_50_) were estimated by regression curves. The data were tested for normality and homogeneity of variance using the Kolgomorov-Smirnov and Levene tests, respectively. Significant differences (p < 0.05) between the tested concentrations and the controls were performed using a one-way ANOVA (with Dunnett’s post hoc) for parametric data and Kruskal-Wallis test (with Dunn’s post hoc) for non-parametric data.

## Results

### Chemical and HPLC analysis

The HPLC analysis revealed that VLX degraded very slowly, under test conditions, over the course of days (Figure S1 and Table S1). At 168 h, a decrease of only ~7% was observed in 16 mg/L of VLX solution (Table S1).

### FET test

**Figure 1 -:**
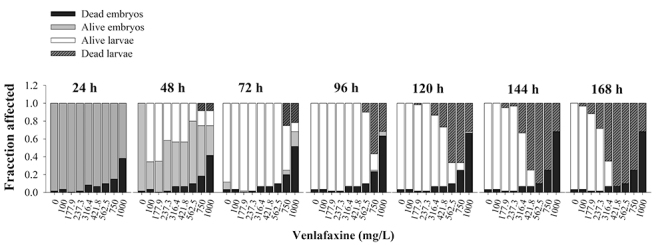
Overview of zebrafish embryo toxicity test after 168 h of exposure to venlafaxine (VLX). The proportion of eggs and non-hatched embryos that died is represented by black bars; the proportion of embryos that stayed alive but did not hatch is presented as grey bars; those that hatched as white bars, and the proportion of embryos that died after hatching as spotted, dashed dark grey bars.

The VLX exposure inhibited embryo development, resulting in mortality after 24 h at concentrations of 316.4, 421.8, 562.5, 750 and 1000 mg/L. Embryo mortality increased in a time-dependent manner (Figure 1). After 144 and 168 h of VLX exposure, 100% of mortality was observed in the higher tested concentrations (562.5, 750.0 and 1000.0 mg/L). The median lethal concentration (LC_50_) was determined after 144 and 168 h of VLX exposure. The sensitivity of embryos and larvae to VLX increased with the exposure time. At 168 h of VLX exposure, we observed a lower LC_50_ (274.1 mg/L, R^2^ = 0.98) compared to the LC_50_ obtained at 144 h (317.5 mg/L, R^2^ = 0.97).

**Figure 2 -:**
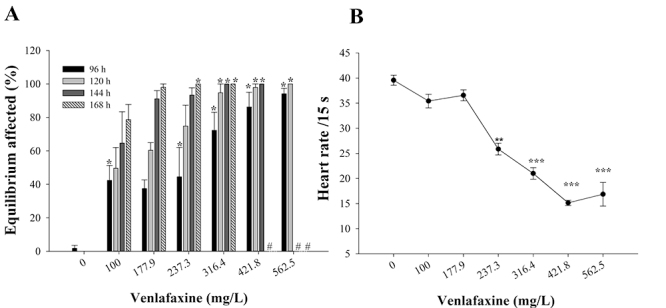
Sub-lethal effects on zebrafish embryo/larvae: (**A**) lack of equilibrium after 168 h of venlafaxine (VLX) exposure, and (B) reduction of embryos’ heart rate after 48 h of VLX exposure. Mean values ± standard error. Asterisk indicates significant differences in relation to the control group: (*) p<0.05, (**) p<0.01 and (***) p<0.001.

Additionally, VLX significantly affected the development of zebrafish embryos, causing sub-lethal effects: premature hatching, lack of equilibrium and reduced heart rate. In the two lower tested concentrations (100.0 and 177.9 mg/L), ~65 % of the exposed embryos hatched at 48 h, compared to ~33% of hatching in the control group (p<0.05) (Table 1). Lack of equilibrium was detected after 96, 120, 144 and 168 h of VLX exposure (Figure 2A). At concentrations of 237.3 (168 h) 316.4 (144 h), 421.8 (144 h) and 562.5 (120 h) mg/L of VLX, all surviving animals presented lack of equilibrium (Figure 2A). After 48 h of VLX exposure (237.3, 316.4, 421.8 and 562.5 mg/L), there was a significant reduction in zebrafish embryos’ heart rate, when compared to the control group (p<0.001) (Figure 2B). The two highest concentrations of VLX (750 and 1000 mg/L) were not considered in Figure 2 due to their high mortality rates.

**Table 1 -:** Hatching (%) for zebrafish embryos during 72 h of VLX exposure ± standard error. (*) p<0.05.

Venlafaxine (mg/L)	Hatching (%) ± Standard error	
	Time (h)	
	48	72
Control	33.3 ± 8.8	91.32 ± 6.3
100	65.53 ± 13.6*	96.49 ± 3.5
177.9	65 ± 5.7*	98.3 ± 1.6
237.3	41.67 ± 8.3	98.3 ± 1.6
316.4	43.3 ± 16.9	93.3 ± 4.4
421.8	43.3 ± 14.5	93.3 ± 3.3
562.5	20 ± 7.6	90 ± 0
750	25 ± 13.2	75 ± 7.6
1000	25 ± 13.2	31.67 ± 18.7

Other developmental changes evaluated during embryo development, such as egg coagulation, lack of otolith formation, delay in development, lack of eye and/or body pigmentation, lack of somite formation, lack of heartbeat, oedemas, spine malformation and swim bladder inflation, did not exhibit significant differences between treated and control groups (data not shown).

### Biomarkers in larvae

**Figure 3 -:**
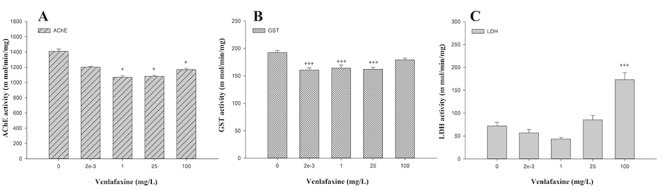
VLX effects on zebrafish embryo enzymatic activities after 168 h of exposure: AChE (**A**), GST (**B**) and LDH (**C**). Mean values ± standard error. Asterisk indicates significant differences in relation to the control group: (*) p<0.05 and (***) p<0.001.

VLX also affected the enzymatic activity of exposed organisms. After 168 h of exposure, AChE activity decreased in zebrafish at concentrations of 1, 25 and 100 mg/L, when compared to the control group (p < 0.05). Although not significant, we also observed a decrease in AChE activity in zebrafish larvae exposed to 0.002 mg/L of VLX (Figure 3A). The GST enzyme presented significantly decreased activity in fish exposed to 0.002; 1 and 25 mg/L (but not in fish exposed to 100 mg/L) of VLX (Figure 3B). In the LDH assay, we observed a significant increase in enzymatic activity only in larvae exposed to the highest concentration of VLX (100 mg/L), compared to the control (p < 0.05) (Figure 3C).

### Adult acute test

During the 96 h of VLX exposure, we did not observe any mortality in any of the concentrations tested. We believe that the LC_50_ is probably higher than 100 mg/L, and therefore, from an environmental and ecotoxicological perspective, it was not pertinent to delimit this value for adult zebrafish.

### Biomarkers in adults

**Figure 4 -:**
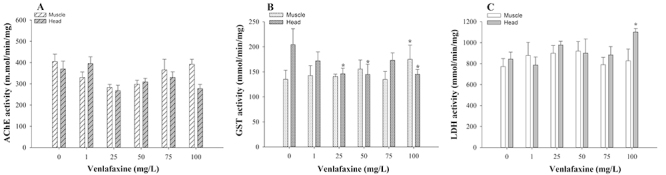
VLX effects on adult zebrafish enzymatic activities after 168 h of exposure: AChE (**A**), GST (**B**) and LDH (**C**) in head and muscle samples. Mean values ± standard error. Asterisk indicates significant differences in relation to the control group: (*) p<0.05.

No significant differences in AChE activity (induction or inhibition) were observed between the exposed animals, at any concentration tested (1, 25, 50, 75 and 100 mg/L of VLX), and the control group (Figure 4A). On the other hand, GST and LDH enzymatic activities were significantly altered at some of the VLX concentrations evaluated. GST activity was significantly inhibited (p<0.05) in head samples of organisms exposed to 25, 50 and 100 mg/L of VLX (Figure 4B). In muscle samples, GST enzymatic activity increased only in animals exposed to the highest concentration of VLX (100 mg/L), in comparison to the control group (p < 0.05) (Figure 4B). For LDH analysis, we observed a significant increase in enzymatic activity only in head samples exposed to 100 mg/L of VLX, compared to the control group (p < 0.05) (Figure 4C).

### Genotoxicity assessment in adults

After 96 h of VLX exposure, the comet assay showed no significant differences between the tested and control groups regarding the percentage of DNA fragmentation in fish erythrocytes (Table 2). In the same way, the micronucleus test did not identify significant differences in the number of MNs and NAs between the exposed groups and the control (Table 3).

**Table 2 -:** DNA fragmentation in peripheral erythrocyte of adult fish after VLX acute exposure. Values represented per mean ± standard error. MD = moderate damage; HD = high damage.

Venlafaxine (mg/L)	DNA fragmentation	
	MD	HD
Control	11 ± 3.1	1.5 ± 1.2
1	13.9 ± 5.0	0.52 ± 0.26
25	13.9 ± 2.22	3.66 ± 3.67
75	15.08 ± 1.66	5.58 ± 2.46
100	14.3 ± 1.84	3.3 ± 1.31

**Table 3 -:** Micronuclei and nuclear abnormalities in peripheral blood erythrocyte of adult fish after VLX acute exposure. Values represented per mean ± standard error.

Venlafaxine (mg/L)	Nuclear alteration					
	Micronuclei	Bud	Binucleated	Blebbed	Lobed	Notched
Control	0	0.0083 ± 0.0001	0.0027 ± 0.0001	0.005 ± 0.0002	0.01 ± 0.0004	0.005 ± 0.0002
1.0	0.019 ± 0.0005	0.0083 ± 0.0001	0.0	0.2 ± 0.07	0.01 ± 0.01	0.05 ± 0.02
25.0	0.007 ± 0.004	0.007 ± 0.004	0.003 ± 0.001	0.013 ± 0.001	0.0	0.008 ± 0
75.0	0.014 ± 0	0.011 ± 0	0.003 ± 0	0.13 ± 0.003	0.008 ± 0.0	0.022 ± 0
100.0	0.027 ± 0.0008	0.03 ± 0.0005	0.0	0.0	0.0	0.005 ± 0.0001

## Discussion

VLX acute exposure has the potential to affect different endpoints in zebrafish embryos/larvae and adults. Below 100 mg/L, embryos/larvae and adults showed similar sensitivity to VLX, which did not significantly increase mortality compared to controls. For the FET test, we chose very high concentrations to find the LC_50._ As a result, mortality rates increased in long exposure, following a concentration dependent relationship (LC_50_-144h = 317.5 and LC_50_-168h = 274.1), as observed for other molecules (Freitas et al., 2017). VLX has been shown to bioaccumulate in *Misgurnus anguillicaudatus* fish, thus indicating that long exposures can lead to increased physiological alterations in the fish, causing metabolic disturbances (Qu et al., 2019).

Hatching is a critical period of zebrafish embryo development and has been widely used as an endpoint in fish early life stage tests. In this study, early hatching was observed in embryos exposed to VLX for 48 h at 100 and 177.9 mg/L. VLX exposure probably caused changes in fish development, which triggered premature hatching. Other studies also indicate that antidepressants can alter the hatch rate in *Danio rerio* (de Farias et al., 2019; Franco et al., 2019). Here, the sub-lethal effect of VLX exposure on equilibrium changes has been described for the first time in zebrafish larvae. A recent study in *Daphnia magna* also identified locomotor pattern changes due to VLX exposure in similar concentrations to ours (immobility EC_50_ of 141.28 mg/L) (Di Poi et al., 2018). Changes in fish equilibrium are a recurrent sub-lethal effect in fish exposed to serotonin reuptake inhibitors (de Farias *et al*., 2019; Oliveira et al., 2021), possibly because this neurotransmitter is related to fish excitatory regulation, stress, anxiety, and behaviour (Herculano and Maximino, 2014). Although lack of equilibrium has also been associated with non-inflated swimming bladders (Lindsey et al., 2010), this phenomenon was very rare in our exposed larvae and did not explain the phenotype.

A reduction in the embryos’ heart rate was another sub-lethal effect identified in zebrafish embryos after 48 h of VLX exposure (237.7; 316.4; 421.8 and 562.5 mg/L). Although VLX does not induce mortality in those concentrations in the first days, this drug may cause physiological changes that compromise the survival of animals over a longer exposure time. To date, there are no studies showing changes in heart rate induced by VLX exposure, and we have identified such sublethal effects here for the first time.

Taken together, our biochemical data suggest that VLX exposure can cause biochemical changes in important pathways related to neurotransmission (AChE), cell detoxifying enzyme (GST) and energy metabolism/biotransformation (LDH) in *Danio rerio* (Lionetto et al., 2013; Rodrigues et al., 2015). Deregulation of AChE activity has been used as a biomarker to study toxic effects in the nervous system (Lionetto *et al*., 2013). VLX inhibits AChE activity in larvae exposed to 1, 25 and 100 mg/L. Of interest, fluoxetine, another antidepressant drug that acts in the same pathway as VLX (inhibition of serotonin reuptake), also altered AChE activity in zebrafish larvae exposed for 120 h to concentrations similar to ours (1; 10 and 100 mg/L) (Pan et al., 2018). Our results indicate that VLX is neurotoxic to zebrafish larvae in concentrations ≥ 1 mg/L. On the other hand, AChE activity was not impaired in zebrafish adults exposed to concentrations similar to embryos/larvae. One hypothesis is that this difference in AChE perhaps indicates a different sensitivity depending on the zebrafish life stage. In the early stages, the brain is in development, and that is why these cells are more sensitive, resulting in increased AChE.

GST is a phase II metabolic enzyme involved in the breakdown of xenobiotics, promoting easier and faster elimination of environmental pollutants (Carletti et al., 2008). We observed a reduction in GST activity both in zebrafish larvae and adults (head) after exposure to VLX, thus suggesting that this drug may cause changes in cell detoxification pathways (Huber et al., 2008). VLX exposure also caused GST inhibition in mouse neurons (Abdel-Wahab and Salama, 2011). Additionally, the downregulation of the *gstp2* gene has been observed in zebrafish embryos exposed for 96 h to 0.3 and 30 µg/L of VLX (Hodkovicova et al., 2020). Since GST acts on detoxification pathways, its inhibition could lead to a greater susceptibility to oxidative stress. Exposure to VLX probably interfered in the xenobiotic elimination processes of cells in zebrafish larvae and adults (Katou et al., 2019). It is important to note that the GST enzyme is part of an integrated defence system, whose efficiency depends on the combined action of several enzymes (Chatterjee and Gupta, 2018). Therefore, for a more conclusive result, other endpoints related to xenobiotic metabolism, such as detoxifying enzymes, would be recommended. 

LDH activity increased significantly after exposure to 100 mg/L of VLX in both larvae (168 h) and adults (96 h), showing a similar physiological response in different life stages of zebrafish. Studies indicate that increased LDH activity, after exposure to contaminants, is directly related to the production and supply of additional energy to the cell to deal with the toxic effects caused by these substances (Rodrigues et al., 2015). In higher concentrations of VLX, the increased activity of LDH may indicate a more frequent use of anaerobic pathways to obtain energy, in comparison to the aerobic way (Levesque et al., 2022; Li et al., 2011). Juvenile rainbow trout (*Oncorhynchus mykiss*) also presented enhanced LDH activity after exposure to VLX for seven days at 0.2 and 1 µg/L (Best et al., 2014). Other antidepressants also altered the activity of LDH in zebrafish, such as carbamazepine and bupropion (da Silva Santos et al., 2018; Franco et al., 2019). 

The genotoxicity experiments (micronucleus test and alkaline comet assay) did not reveal significant DNA damage (fragmentation and abnormalities) in the erythrocytes of zebrafish adults exposed to different concentrations of VLX. Our results suggest that VLX has no genotoxic or mutagenic action in zebrafish adults. VLX also presents no genotoxicity in humans under therapy (Ahmadimanesh et al., 2019). Moreover, studies with other antidepressants, such as doxepin, escitalopram, fluoxetine, duloxetine and sertraline, did not show genotoxic effects (Pereira et al., 2009; Cobanoglu et al., 2018; Istifli et al., 2018; de Farias et al., 2020). Since the use of VLX is widespread around the world, and its detection has been consistently tested in different aquatic ecosystems, the verification of non-genotoxic effects in the zebrafish model represents, from an ecotoxicological perspective, a positive aspect for the survival of aquatic communities.

An interesting study carried out by Mehdi et al. (2019) demonstrated that zebrafish exposed to VLX at 1.0 µg/L and 32⁰C underwent an increase in catalase activity and other metabolic physiological parameters, compared with exposures at 1.0 µg/L and 27⁰C, indicating a deleterious effect on the antioxidant defense. In a scenario of global warming, increased water temperature has an influence on elevated metabolism and energy production, causing oxidative stress. The authors pointed out that increased temperature caused more significant changes than VLX exposure. 

In conclusion, this study showed the adverse effects of VLX on *Danio rerio* at different life stages, from embryos to adults. In embryos, acute exposure (168 h) caused several disturbances, such as mortality leading to LC_50_ of 274.1 mg/L, decreasing heartbeat, premature hatching, alterations in equilibrium and changes in enzymatic biomarkers (AChE, GST and LDH). In adults, VLX caused alterations in GST and LDH activities, not exercising genotoxic effects. Short-term exposure to this antidepressant can negatively affect fish survival in multiple ways. Considering the continuous flow of VLX to the environment, and its moderate persistence in the water (Shen et al., 2018), additional chronic ecotoxicological studies using very low concentrations and sensitive endpoints are required, to explore the toxicity mechanisms of VLX more thoroughly.

## Supplementary material

Table S1 - Concentrations of venlafaxine and percentage of recovery from the stock solution (nominal concentration of 16 mg/L). Standard deviation in brackets.

Figure S1 - Calibration curves of venlafaxine.
