# Supplementary material for: Multi-level toxicity assessment of the antidepressant venlafaxine in embryos/larvae and adults of zebrafish (Danio rerio)
Source: Genet Mol Biol. 2023 Sep 8;46(3):e20220377. doi: 10.1590/1678-4685-GMB-2022-0377 (PMC10494572; doi:10.1590/1678-4685-GMB-2022-0377)
Supplement: Table S1 - [file 1415-4757-GMB-46-3-e20220377-s1.pdf]

Genetics and Molecular Biology
https://doi.org/10.1590/1678-4685-GMB-2022-0377

Supplementary Material to "Multi-level toxicity assessment of the antidepressant venlafaxine in embryos/larvae and adults of zebrafish (*Danio rerio*)"

Table S1 - Concentrations of venlafaxine and percentage of recovery from the stock solution (nominal concentration of 16 mg/L). Standard deviation in brackets.

Time (days)	Fish conditions*		
	Concentration (mg/L)		% of recorvery
Day 0	16.17	(0.081)	**100**
Day 1	16.37	(0.019)	**102.33**
Day 2	15.86	(0.44)	**98.75**
Day 3	15.86	(0.48)	**96.86**
Day 4	15.82	(0.43)	**96.63**
Day 5	15.70	(0.31)	**95.92**
Day 6	15.55	(0.33)	**95.01**
Day 7	15.31	(0.25)	**93.52**

*: Fish conditions: 26.0 ± 1°C, photoperiod 12 h light: 12 h dark, light intensity of 32 lux.
