# Supplementary material for: Multi-level toxicity assessment of the antidepressant venlafaxine in embryos/larvae and adults of zebrafish (Danio rerio)
Source: Genet Mol Biol. 2023 Sep 8;46(3):e20220377. doi: 10.1590/1678-4685-GMB-2022-0377 (PMC10494572; doi:10.1590/1678-4685-GMB-2022-0377)
Supplement: Figure S1 - [file 1415-4757-GMB-46-3-e20220377-s2.pdf]

Genetics and Molecular Biology
https://doi.org/10.1590/1678-4685-GMB-2022-0377

Supplementary Material to "Multi-level toxicity assessment of the antidepressant venlafaxine in embryos/larvae and adults of zebrafish (*Danio rerio*)"

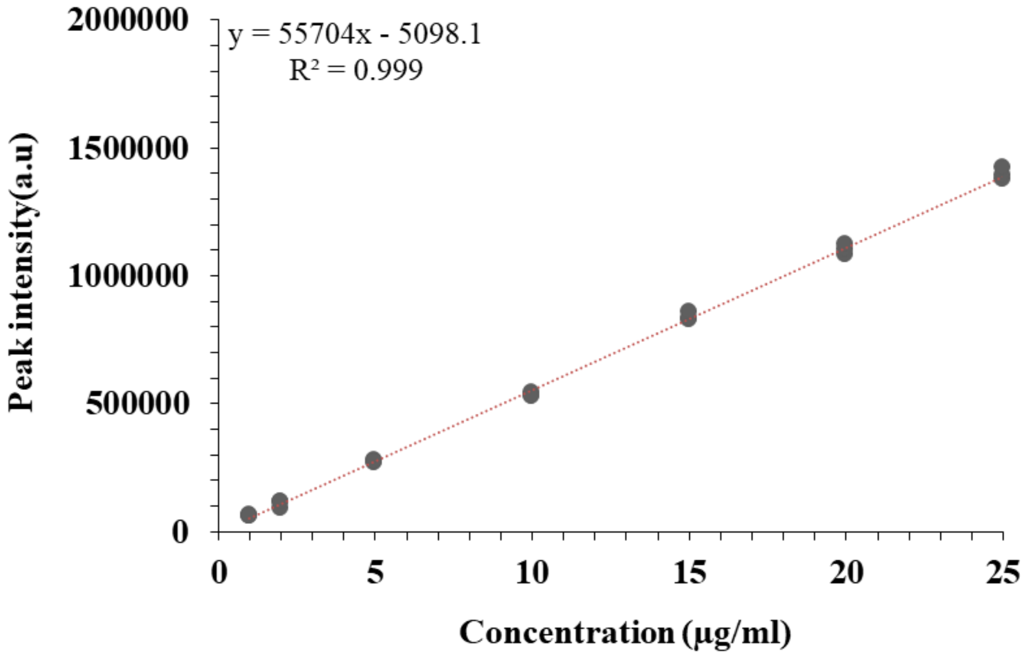

Figure S1 - Calibration curves of venlafaxine.
